# Connectivity-based parcellation of the amygdala and identification of its main white matter connections

**DOI:** 10.1038/s41598-023-28100-6

**Published:** 2023-01-24

**Authors:** Josue M. Avecillas-Chasin, Simon Levinson, Taylor Kuhn, Mahmoud Omidbeigi, Jean-Philippe Langevin, Nader Pouratian, Ausaf Bari

**Affiliations:** 1 Department of Neurosurgery, University of Nebraska Medical Center, 988437 Nebraska Medical Center, Omaha, NE 68198-8437 USA; 2 David Geffen School of Medicine, University of California Los Angeles, Los Angeles, CA USA; 3 Department of Psychiatry and Biobehavioral Sciences, David Geffen School of Medicine at UCLA, University of California, Los Angeles, CA USA; 4 Department of Neurosurgery, David Geffen School of Medicine, University of California, Los Angeles, CA USA; 5 Neurosurgery Service, VA Greater Los Angeles Healthcare System, Los Angeles, CA USA; 6 Department of Neurological Surgery, UT Southwestern Medical Center, Dallas, TX USA

**Keywords:** Emotion, Neural circuits

## Abstract

The amygdala plays a role in emotion, learning, and memory and has been implicated in behavioral disorders. Better understanding of the amygdala circuitry is crucial to develop new therapies for these disorders. We used data from 200 healthy-subjects from the human connectome project. Using probabilistic tractography, we created population statistical maps of amygdala connectivity to brain regions involved in limbic, associative, memory, and reward circuits. Based on the amygdala connectivity with these regions, we applied k-means clustering to parcellate the amygdala into three clusters. The resultant clusters were averaged across all subjects and the main white-matter pathways of the amygdala from each averaged cluster were generated. Amygdala parcellation into three clusters showed a medial-to-lateral pattern. The medial cluster corresponded with the centromedial and cortical nuclei, the basal cluster with the basal nuclei and the lateral cluster with the lateral nuclei. The connectivity analysis revealed different white-matter pathways consistent with the anatomy of the amygdala circuit. This in vivo connectivity-based parcellation of the amygdala delineates three clusters of the amygdala in a mediolateral pattern based on its connectivity with brain areas involved in cognition, memory, emotion, and reward. The human amygdala circuit presented in this work provides the first step for personalized amygdala circuit mapping for patients with behavioral disorders.

## Introduction

The amygdala is a heterogenous nuclear complex located in the mesial temporal lobe. The amygdala has been associated with fear and aversion^1^. However, recent evidence has extended the role of the amygdala to other functions such as emotional memory, learning, reward-based and goal-directed behaviors^1,2^. The amygdala is also involved in behavioral disorders including aggression, addiction, anxiety, substance abuse, epilepsy, and post-traumatic stress disorder (PTSD)^2–6^. The functional characterization of amygdala circuitry in humans is foundational for the understanding of these neuropsychiatric diseases and for developing new neuromodulatory treatments targeting the amygdala circuit^7–9^. The functional and structural configuration of the amygdala circuit has been extensively described in the literature but mainly focused on the parcellation of the amygdala with still paucity of descriptions of its main white matter (WM) bundles^10–16^. Solano-Castiella et al., segmented the amygdala based on diffusion anisotropy. They characterized a medial segment with anterior–posterior fiber orientation and a lateral segment with a dorsoventral orientation that was consistent with post-mortem brain studies^13^. The limited number and the lack of connectivity information of these segments would limit the interpretation of these findings in terms of the functional model of the amygdala circuit. Bach et al., on the other hand, parcellated the amygdala in two clusters using connectivity with the cortex. The deep cluster had stronger connectivity with the temporal pole and the superficial cluster had stronger connectivity with the orbitofrontal cortex OFC^12^. Even though these two cortical regions have connections with the amygdala, the limited number of cortical regions and the lack of integration with subcortical structures would restrict the findings to the corticoamygdalar arm of the amygdala circuit. Saygin et al., used probabilistic tractography to characterize four segments of the amygdala including the lateral, basal, central, and medial-cortical nuclei^11^. These authors used the amygdala as seed and several cortical and subcortical brain regions as targets for fiber tracking. These authors used a priori anatomical knowledge to properly classify well known connections of the amygdala and obtain the parcellations based on this information. Although this approach was more comprehensive in terms of amygdala connectivity with cortical and subcortical regions, still there was no description of the WM bundles that connect these amygdala segments with the rest of the brain.

In general, the functional model of the amygdala has been described as the integration of information from limbic, associative, visual, auditory, somatosensory, reward, and memory areas of the brain to tailor adequate behavioral responses according to the context and the nature of the stimuli^1,6,17,18^. In this context, using a more targeted approach, with the associative and limbic circuits to parcellate the amygdala, may provide a more functionally meaningful parcellation. In this work, we aimed to combine connectivity-based parcellation of the human amygdala with the identification of its main WM bundles. To this aim, we used local patterns of connectivity of the amygdala with specific brain regions related to behavior to delineate sub-regions within the amygdala. We hypothesize that using specific brain regions involved in the modulation of human behavior would provide a meaningful parcellation of the human amygdala consistent with previous work. Moreover, using probabilistic tractography, we investigated how these different amygdala sub-regions are connected with the rest of the brain. This will provide a detailed mapping of the human amygdala circuitry and its main hubs.

## Materials and methods

### Data acquisition

The first dataset was obtained from the publicly available WU-Minn HCP 1200 subjects data release repository^19^. The scanning protocol was approved by Human Research Protection Office (HRPO), Washington University (IRB# 201 204 036). The participants included in the HCP 1200 Subjects data release provided written informed consent as approved by the Washington University IRB and the study was carried out in accordance with the Declaration of Helsinki. From this repository, 200 non-twin subjects were randomly selected (age 29 SD 4, men 48% and women 52%). Of the 200 total subjects, three subjects were excluded due to incomplete diffusion MRI data and 29 subjects were excluded due to missing data during the tractography classification process. The results are based on the remaining 168 subjects. The data were acquired in a modified Siemens 3T Skyra scanner with a customized protocol^20^. The T1-weighted MRI has an isotropic spatial resolution of 0.7 mm, and the dMRI data have an isotropic spatial resolution of 1.25 mm. The multi-shell dMRI data were collected over 270 gradient directions distributed over three b-values (1000, 2000, 3000 s/mm2). For each subject, the multi-shell dMRI data were collected with both L/R and R/L phase encodings using the same gradient table, which were then merged into a single copy of multi-shell dMRI data after the correction of distortions with the HCP Preprocessing Pipeline^21^. We used a second independent dataset as a validation step to map the main white matter pathways of the amygdala. This second dataset was obtained from the neuroimaging community sample Nathan Kline Institute-Rockland Sample (NKI-RS)^22^ consisting of twenty-eight subjects (mean age 36 SD 14, men 30%, women 70%). The specifications of the imaging protocol are available at http://fcon_1000.projects.nitrc.org/indi/enhanced/mri_protocol.html. The data of the two datasets were corrected for Eddy current-induced distortion and subject movement^23^.

### Tractography classification

**Figure 1:**
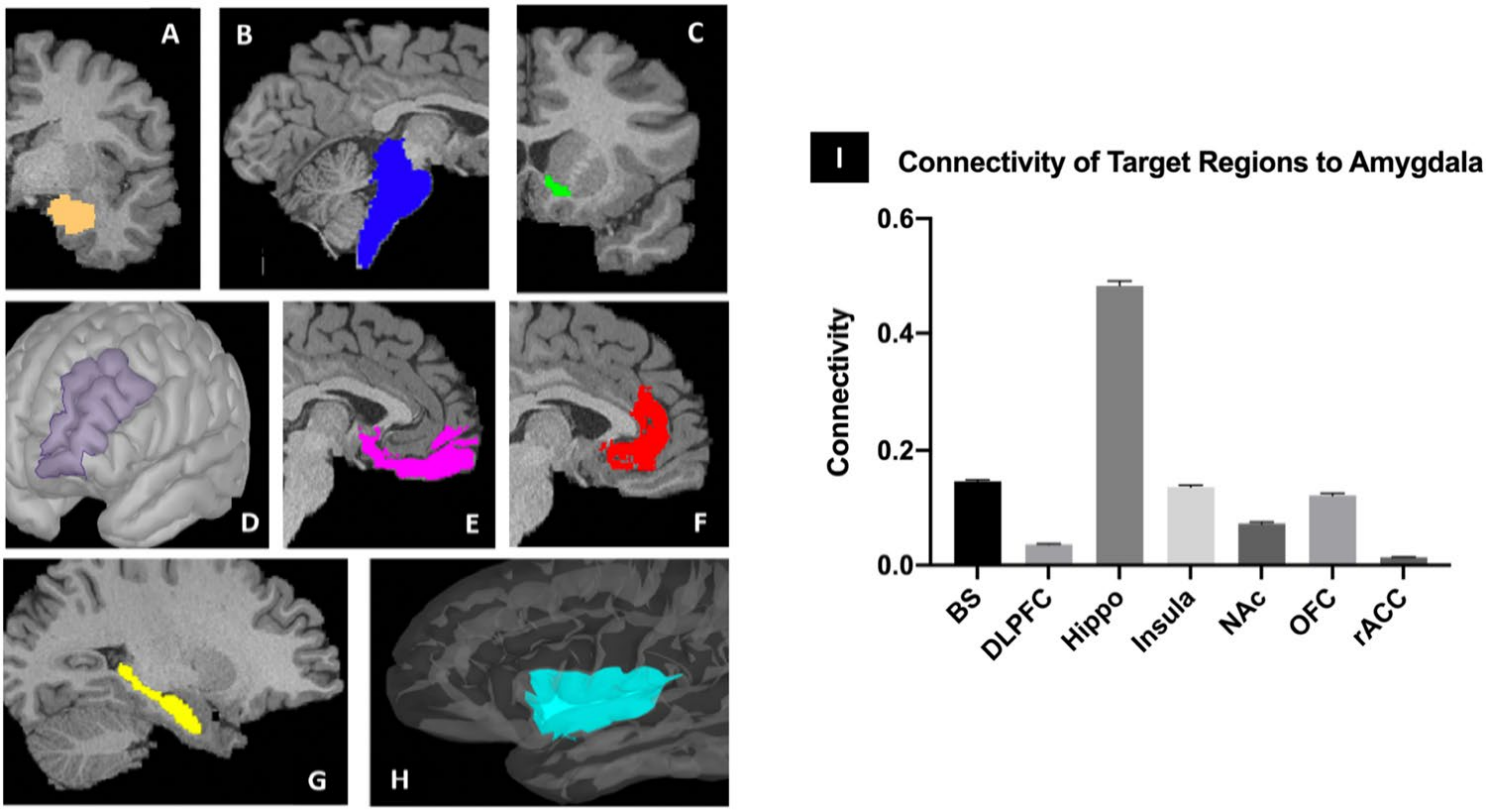
Cortical (**D**, **E**, **F**, **H**) and subcortical (**B**, **C**, **G**) masks of the target brain areas and the amygdala (A, center) from a single subject. (**A**): Amygdala. (**B**): Brainstem (BS). (**C**): Nucleus accumbens (NAc) (**D**): Dorsolateral prefrontal cortex (DLPFC). (**E**): Orbitofrontal cortex (OFC). (**F**): Rostral anterior cingulate cortex (rACC). (**G**): Hippocampus (Hippo). (**H**): Insula^30^. (**I**) Probability of connectivity from the amygdala to each target region. The connection probability of each voxel of the amygdala to each of the seven target regions was averaged over all amygdala voxels and this value then averaged across all subjects. Note that the highest probability of connectivity is to the hippocampus (48%). Thus, 48% of all streamlines from the amygdala to the above targets terminated in the hippocampus.

Probabilistic tractography was performed using FSL’s FMRIB Diffusion Toolbox (probtrackx) with modified Euler streaming^24,25^. Seed and target masks were generated using the Harvard–Oxford subcortical atlas^26^. Seed masks included bilateral amygdala. Target masks included the nucleus accumbens (NAc), brainstem nuclei (BS), hippocampus (HC), dorsolateral prefrontal cortex (DLPFC), insular cortex, orbitofrontal cortex (OFC) and rostral anterior cingulate cortex (rACC)^27–29^. These brain target regions were selected based on its well-known role in human behavior. All tractography was performed in native space between each (right and left) amygdala and the ipsilateral target masks except that the entire BS (left and right side) was used as a target for each amygdala (Fig. 1). All masks were used from the freesurfer individual segmentation including in this database and edited of necessary according to the general definition of the brain target regions. Each target mask was also a termination mask such that tractography was terminated once a streamline entered the target. Ipsilateral white matter masks were used as waypoints. The ventricles and cerebellum were used as exclusion masks. The tractography parameters were as follows: “onewaycondition”, curvature 0.2, 5000 samples, steplength = 0.5, fibthresh = 0.01, distthresh = 1 and sampvox = 0.0 and omatrix2 option, and distance correction. FSL commands were performed on remote servers using Amazon Web Services (AWS, http://aws.amazon.com) EC2 instances running in parallel. Each AWS EC2 instance was an r4.large clone of an Amazon Machine Image running Ubuntu 14.04 with FSL software version 5.0.10. This allowed us to run tractography on all subjects simultaneously in parallel in native diffusion space. FSL bedpostx directories for each subject and the probtrackx output files were stored on an Amazon S3 bucket.

### k-means clustering

**Figure 2:**
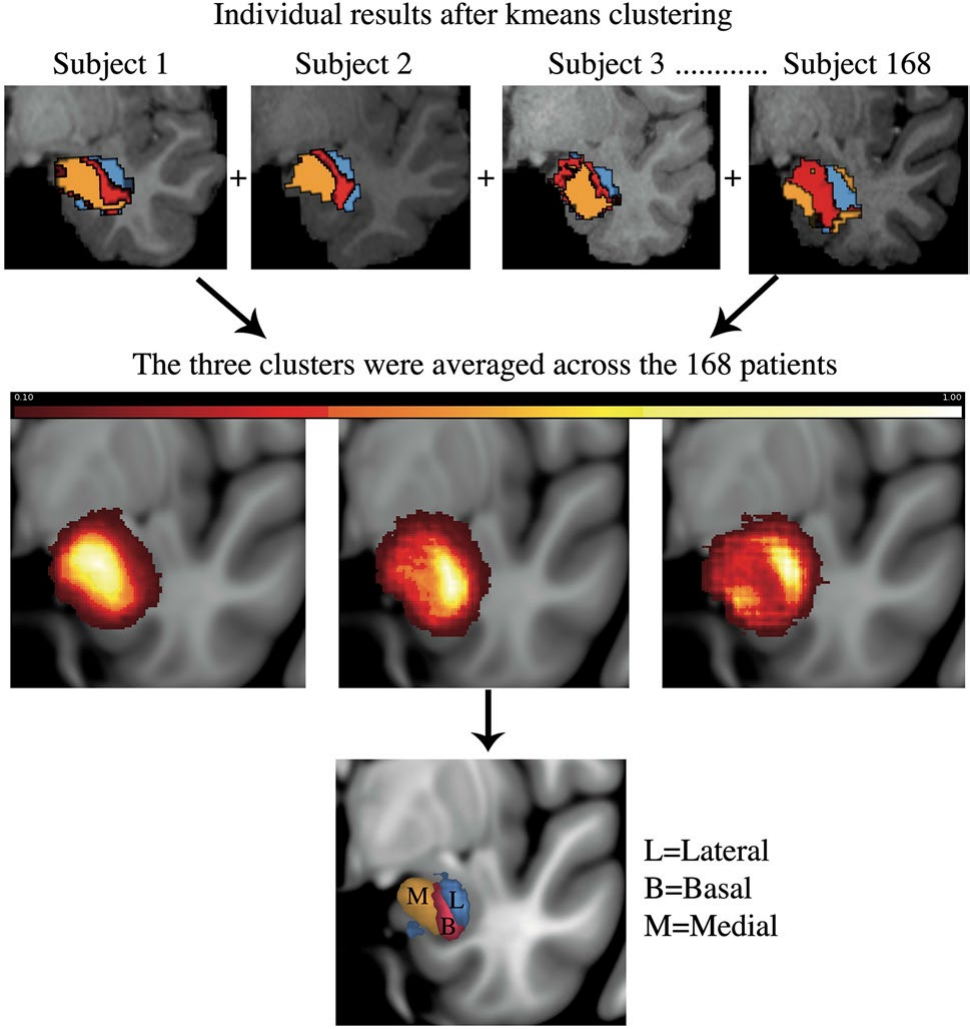
Individual results were normalized to a common MNI space. The clusters were separated and summed across all 168 subjects such that each voxel value in the final cluster represented the number of subjects with the cluster in that location. These group probability maps of the clusters were then thresholded at 50% to optimize the overlap between each other.

The *k-means* clustering algorithm was implemented in Matlab 2015b. This algorithm allows to divide *n* observations (voxels) into *k* groups using a centroid point as reference and clustering its nearest points based on the connectivity similarities of each voxel. First, the connectivity pattern of the amygdala with each of the target brain regions was computed and arranged in the connectivity matrix (matrix 2) between the seed (amygdala) and all points in the target masks. This was used to generate a cross-correlation matrix (CCM) for each subject. Second, to define the voxels within the amygdala mask with a similar connectivity pattern with the brain target regions, the *k-means* clustering algorithm was applied to the CCM with a predefined number of three clusters (*k* = 3) to reflect the gross established anatomical and functional configuration of the amygdala (centromedial, basal, and lateral nuclear groups)^10,31,32^. Third, the connectivity-based clusters of all subjects were normalized to the standard MNI-152 space using linear registration with FLIRT followed by non-linear registration with FNIRT. The clusters were then separated and summed across all subjects to create common cluster maps. In this fashion, each voxel value in the final common cluster represented the number of subjects with the cluster in that location (Fig. 2). These common cluster maps were finally thresholded at 50% to define the structural boundaries between each cluster based on the anatomical similarity with the ground-truth amygdala configuration^31,33^.

### Probability of connectivity of the clusters

We calculated the connection probability of each of the seven brain target regions with the amygdala and each of the amygdala clusters at the individual level^30^. After the tractography classification analysis, we ran the FSL command *proj_thresh* with a threshold of 1250 on each PROBTRACKX output. For each voxel in the seed mask with a value above the threshold, *proj_thresh* calculates the number of samples reaching each of the target masks as a proportion of the total number of seeds. This yielded a separate map of the amygdala for each target with each voxel having a value between 0 and 1 representing the connection probability of that voxel to the given target. To produce an overall probability of connectivity of the seven target brain regions with each amygdala cluster, probabilities were averaged across all voxels within each of the three clusters using *fslstats*. These probabilities were then averaged across all subjects for the left and right amygdala. We then used the one-way ANOVA with a post-hoc Bonferroni correction for multiple comparisons to analyze the differences in connectivity between each of the seven target regions and the three clusters in each hemisphere across subjects. Significance level was set at corrected *p* < 0.05 and we used GraphPad Prism software (San Diego, CA, US).

### Tractography analysis of the clusters

To identify the white matter connections of the three clusters with the rest of the brain, we performed a seed-based tractography analysis on dataset 2 as an anatomical validation step. The imaging data of all subjects, including T1-weighted and diffusion-weighted images (DWI), were pre-processed using FSL tools and PANDA tools^34^. High resolution T1-weighted images underwent skull stripping using BET. We used the DTIFIT tool for diffusion tensor imaging fitting, and BEDPOSTX tool to estimate the probability distribution of at most three fiber populations at every voxel^35,36^. We then registered the DWI data with the anatomical T1, and with the standard MNI-152 space using two-stage linear registration with FLIRT and non-linear registration with FNIRT. Each amygdala population cluster from the previous analysis was transformed to the native space of each subject in this second validation dataset. Probabilistic tractograms were generated seeding from each cluster using the PROBTRACKX tool with the same parameters used as for dataset 1. The tractograms were normalized, thresholded at 95% probability of connectivity, binarized, and summed between subjects using *fslmaths*. These group averaged maps were then thresholded based on the known anatomical features of the white matter connections of the amygdala according to ground-truth anatomical evidence^31,33^.

## Results

**Figure 3:**
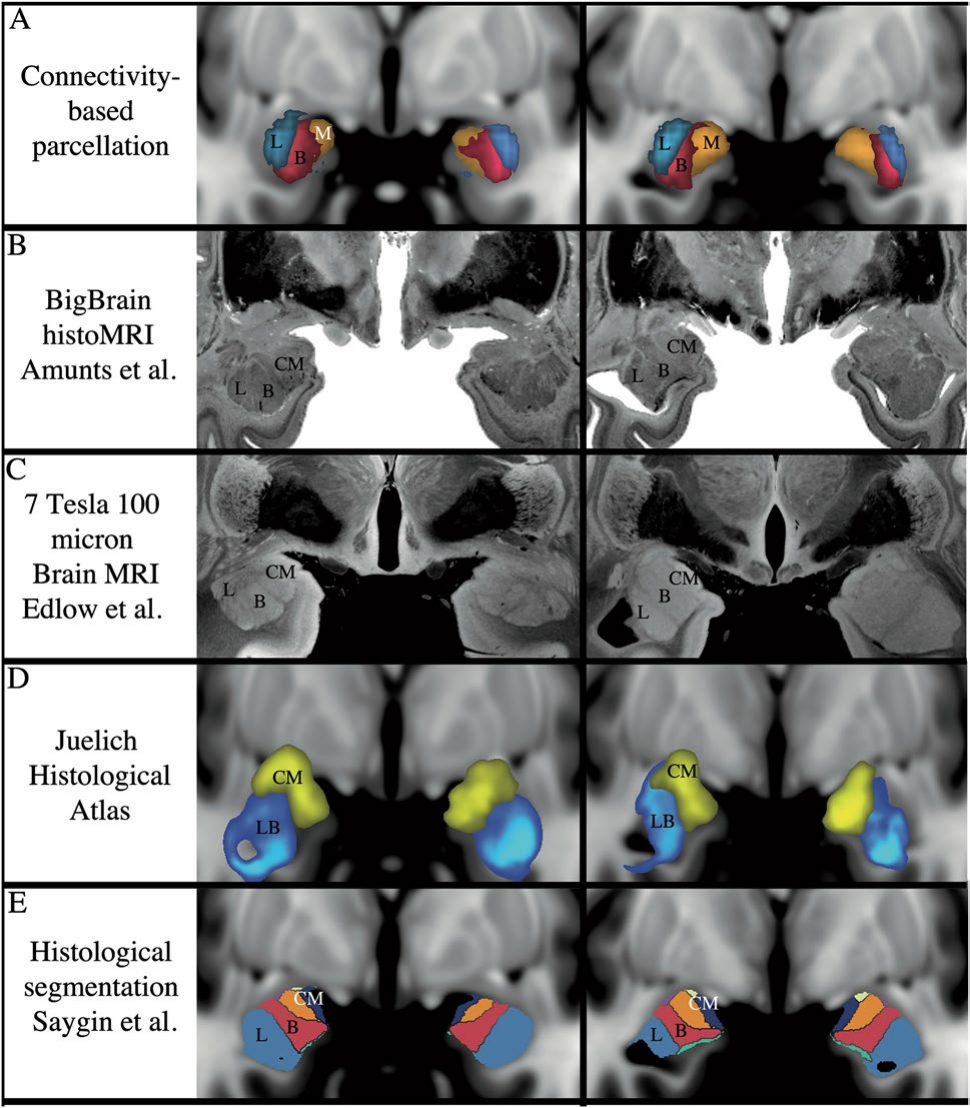
Qualitative comparison of our connectivity-based parcellation with other amygdala atlases^32,37–40^. (**A**) Our results show three clusters in a mediolateral pattern that is consistent with the gross structural configuration of the amygdala described in previous studies (**B** through **E**). L, lateral. (**B**) Basal. M, medial. CM, centromedial.

We performed probabilistic tractography from the amygdala to seven brain target regions. Of these brain regions, the HC and OFC had the highest probability of connectivity to the whole amygdala, followed by the BS and insula, and with the lowest connectivity with the DLPFC, NAc, and rACC (Fig. 1I). Based on differences in connectivity profiles of the amygdala with brain regions involved in reward, memory, limbic, and cognitive functions, we found three spatially contiguous clusters configured in a medial to lateral pattern in 85% of the subjects (Fig. 2). The average volume of the amygdala was 1759 mm^3^, the average volumes of the medial lateral and basal cluster thresholded at 50% were 1218 mm^3^, 821 mm^3^, 867 mm^3^ respectively. We qualitatively compared our results with different brain atlases and other segmentations of the amygdala and our clusters grossly corresponded with the amygdala nuclear groups described in histological studies (Fig. 3)^32,37–40^. The medial cluster corresponded to the central, medial, and cortical nuclei. The basal cluster corresponded to the basal nuclear complex and the lateral cluster corresponded with the lateral nuclei of the amygdala.

**Figure 4:**
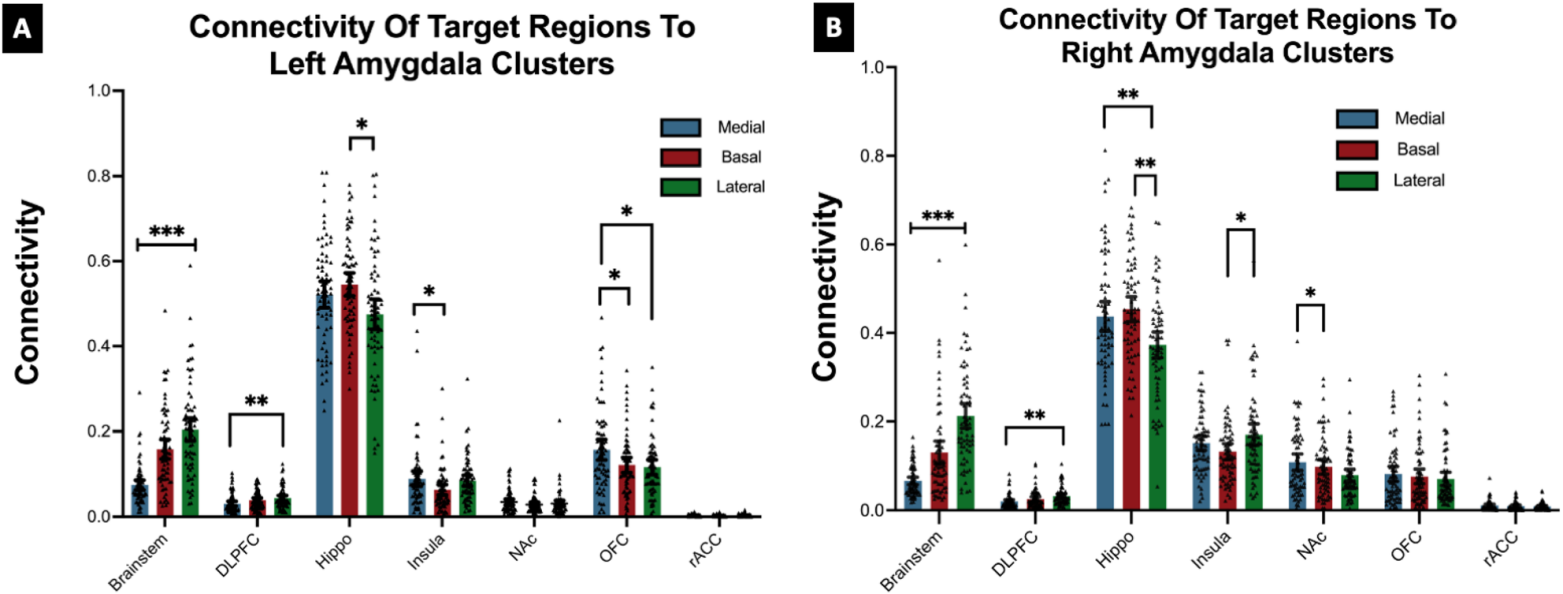
Connectivity (measured by probability of connectivity 0–1, *y* axis) of the seven target regions to each amygdala cluster. DLPFC (dorsolateral prefrontal cortex), Hippo (hippocampus), NAc (nucleus accumbens), OFC (orbitofrontal cortex), rACC (rostral anterior cingulate cortex). *= *p ≤ *0.05, **= *p* ≤ 0.01, ***= *p* ≤ 0.001.

The probability of connectivity of each of the seven brain regions to each cluster are shown in Fig. 4. The most clear and significant patterns of connectivity were as follows: The brainstem showed stronger connectivity with the lateral cluster bilaterally; left: (20.5%) *p* = 0.001, df = 1404, right: (21.2%) *p* =  < 0.001, df = 1406. The DLPFC showed stronger connectivity with the lateral cluster bilaterally; left: (4.5%), df = 1404 *p* = 0.004, right: (3.2%) *p* = 0.004, df = 1406. The HC had stronger connectivity with the basal cluster bilaterally: left: (53.8%), df = 1404 *p* = 0.021, right: (45.4%) *p* =  < 0.000, df = 1406. The insula had stronger connectivity with the medial cluster on the left: (8.9%) *p* = 0.035, df = 1404 and with the lateral cluster on the right: (17%) *p* = 0.021, df = 1406.

**Figure 5:**
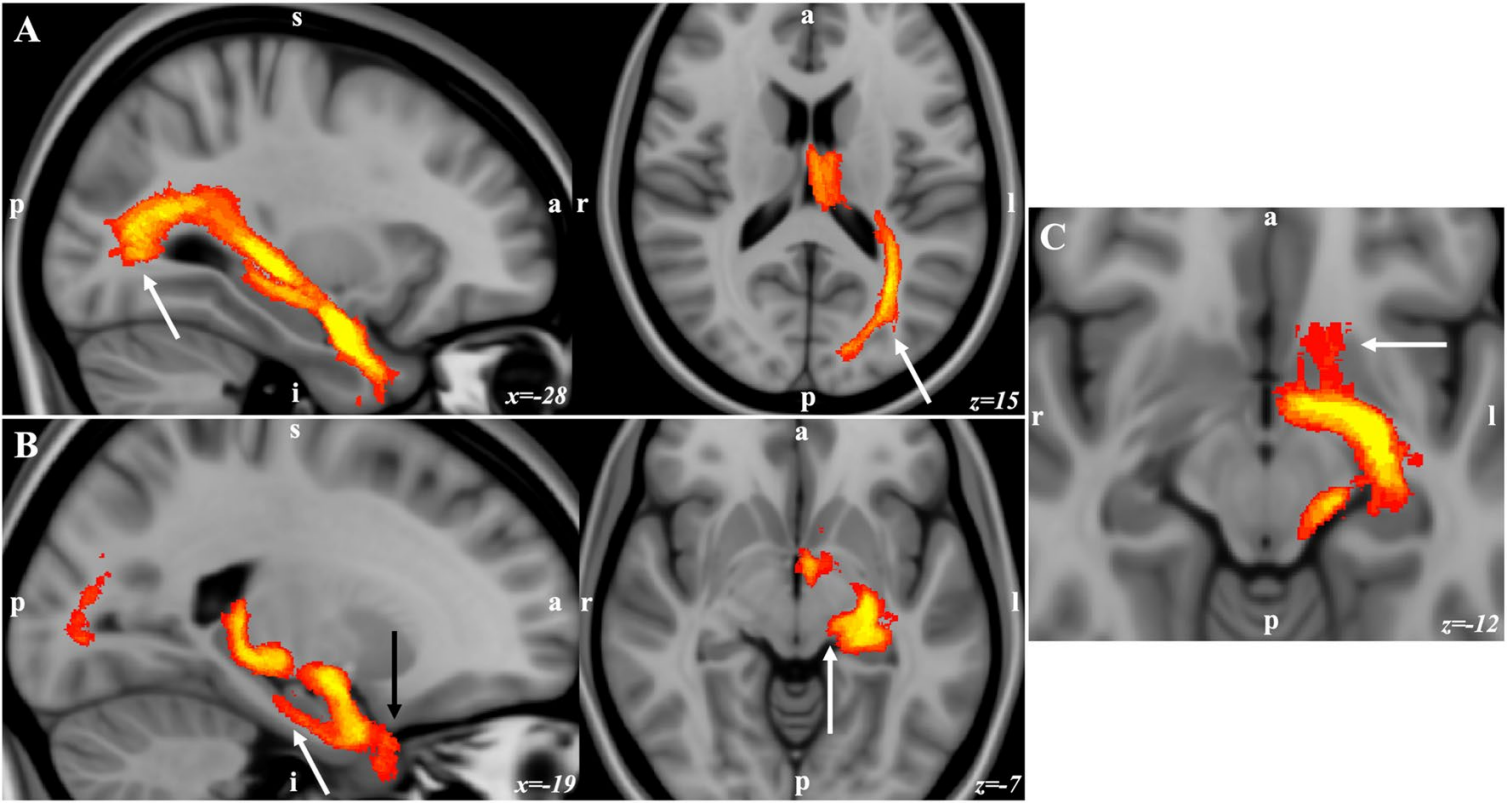
Population connectivity maps of the amygdala clusters. (**A**) The lateral cluster also showed stronger connectivity with sensory areas including parietal, occipital, and posterior temporal areas (arrows) (**B**) The basal and lateral clusters also showed stronger connectivity through the parahippocampal radiation of the cingulate bundle (white arrow in sagittal view), with the temporal pole (black arrow in sagittal view), and through the amygdalotectal pathways with the colliculi. (**C**) The lateral cluster had stronger connectivity through the subcaudate white matter and ventromedial part of the uncinate fascicle with the PFC (arrow).

**Figure 6:**
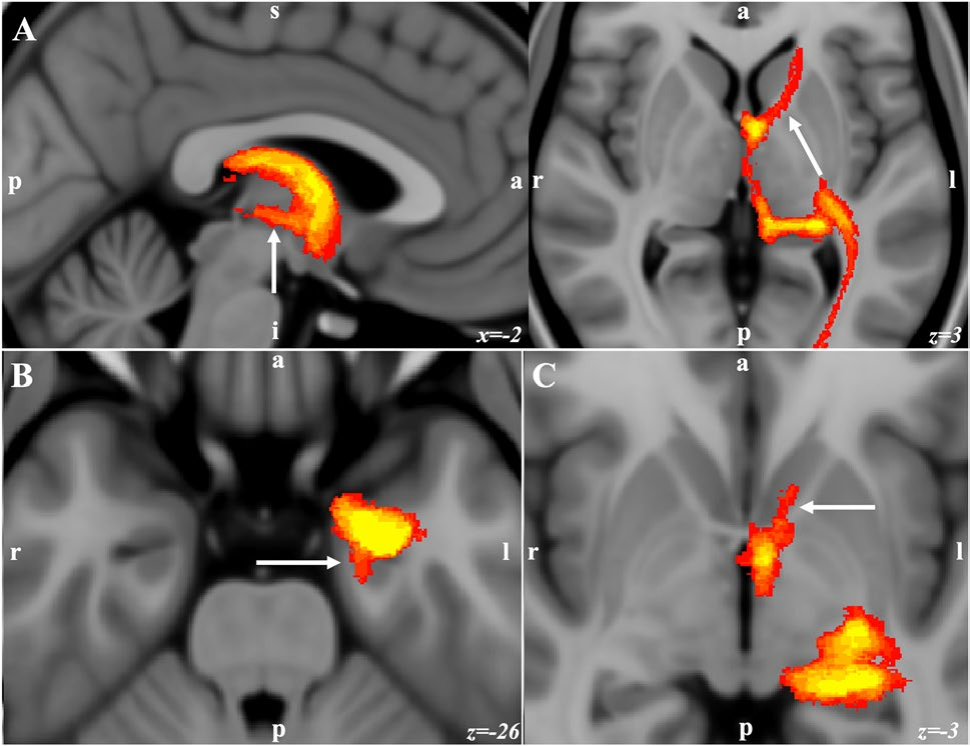
Population connectivity maps of the amygdala clusters. (**A**) The basal cluster had stronger connectivity with the medial thalamus through the inferior thalamic peduncle (arrow in sagittal), and through the anterior limb of the internal capsule with the PFC (arrow in axial view). (**B**) with the HC through the amygdalohippocampal bundle and (arrow). (**C**) with the ventral striatum (arrow in **C**).

**Figure 7:**
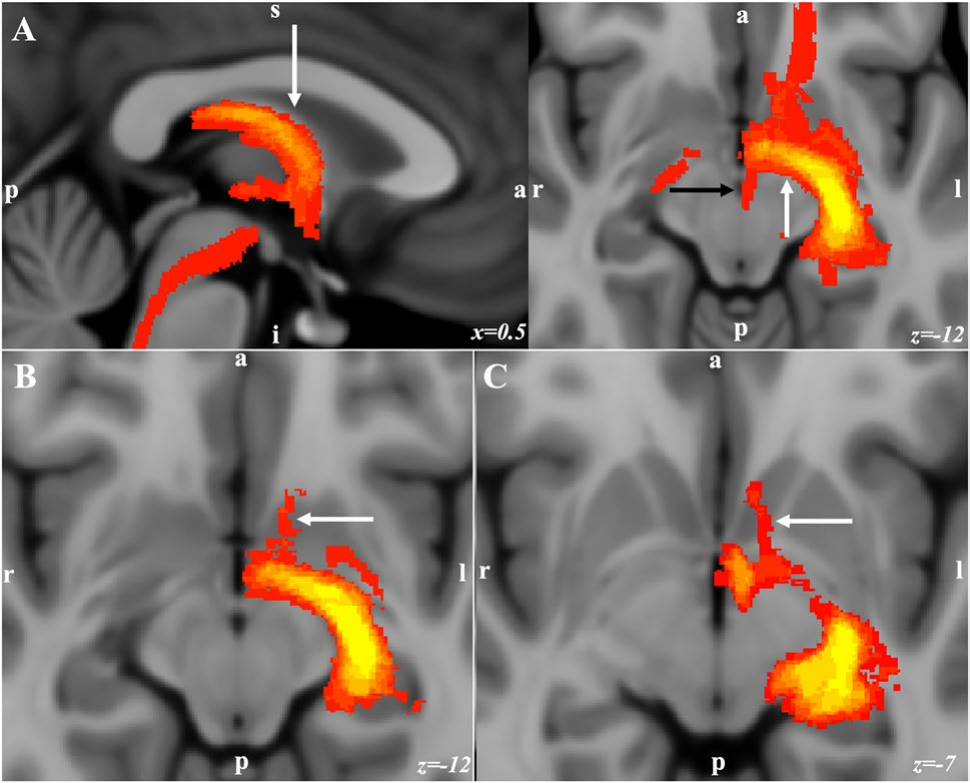
Population connectivity maps of the amygdala clusters. (**A**) The medial and basal clusters showed stronger connectivity through the stria terminalis (arrow in sagittal view) and ventral amygdalofugal pathway (arrow in axial view). These two pathways converged in the BNST, hypothalamus, and septal area. The medial cluster showed unique connectivity with the ventral tegmental area through the medial forebrain bundle (black arrow in axial). (**B**) and (**C**) The medial and lateral cluster showed stronger connectivity with ventral and dorsal areas of the NAc respectively.

**Figure 8:**
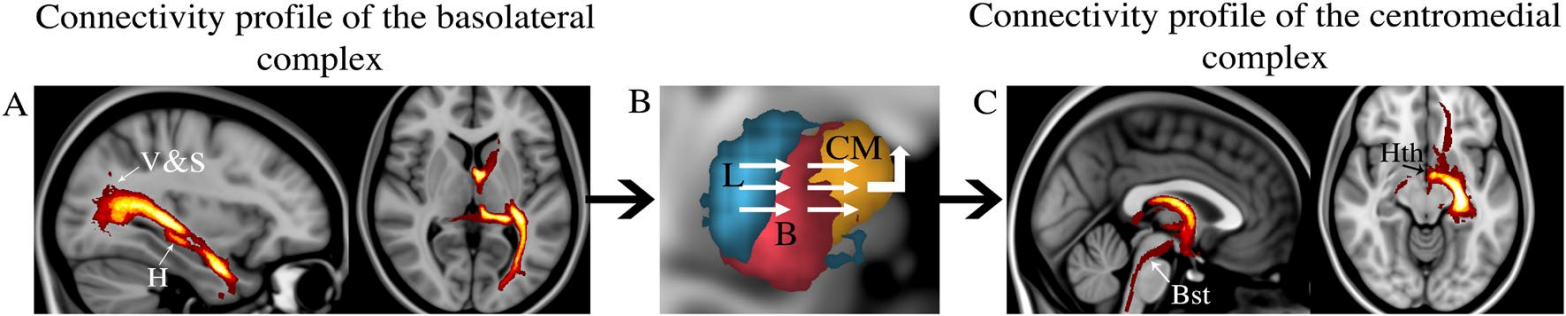
Functional model of the amygdala circuit. (**A**) The basolateral complex (L, B) receives and integrates information [from visual (V), sensory areas, hippocampus (H), and prefrontal cortex]. (**B**) The centromedial complex (CM) translates this information to behavior through the connections with the hypothalamus (Hth) and the brainstem (Bst).

The tractography analysis seeding from the three clusters demonstrated the lateral cluster with strong connectivity with sensory areas including the parietal, occipital, posterior temporal, and superior colliculus. The lateral cluster also had strong connectivity through the uncinate fascicle with the prefrontal cortex (PFC) (Fig. 5). The lateral and basal cluster showed strong connectivity with the temporal pole and cingulate cortex. The basal cluster showed connectivity with the PFC and ventral striatum through the amygdalofugal pathways and internal capsule. This basal cluster also had stronger connectivity with the HC through the amygdalohippocampal bundle, with the medial thalamus through the inferior thalamic peduncle, and with the cingulum bundle (Fig. 6). The medial and basal clusters showed stronger connectivity through the ventral amygdalofugal pathway (VAF) and the stria terminalis (ST). These two pathways converged in the bed nucleus of the stria terminalis (BNST), hypothalamus, and septal area. The medial cluster showed connectivity with the NAc and unique connectivity with the ventral tegmental area (VTA) through the medial forebrain bundle, also involved in reward-related behaviors (Fig. 7). These findings are consistent with the functional model of the amygdala showed in the Fig. 8 with the lateral cluster processing sensory information, lateral and basal clusters processing contextual information from temporal, cingulate, and prefrontal cortex, and hippocampus. Finally, the basal and medial clusters processing the output information for behavioral responses connecting with the hypothalamus, basal forebrain, and brainstem^1,17,41^.

## Discussion

The amygdala circuitry plays a pivotal role in modulating behavior. In this work, we parcellated the amygdala based on its connectivity with specific brain regions involved in different aspects of behavior. This in vivo mapping of the amygdala circuitry showed a parcellation of the amygdala with a medial to lateral pattern and corresponded with the structural organization that has been found in previous histological and tracing studies^14,31,33,42,43^. This three-cluster parcellation scheme was also previously validated by other authors^10,44^ and there is general agreement that the amygdala is subdivided into three groups of nuclei^13,33^. Our medial cluster corresponded to the central, medial, and cortical nuclei. The basal cluster corresponded to the basal nuclear complex and the lateral cluster corresponded to the lateral nuclei of the amygdala. We were also able to delineate the white matter connections of the amygdala including the ventral amygdalofugal pathway, stria terminalis, cingulum bundle, amygdalohippocampal bundle, amygdalotectal bundle, uncinate fasciculus, and external capsule^33^. This configuration of the clusters and their connections with the rest of the brain could give us clues of the role of the amygdala in integrating sensory information to modulate behavior^15,31,33^.

The circuit of the amygdala has been described as follows: the lateral nucleus of the amygdala receives sensory information from cortical and subcortical structures and regulatory information from the PFC. The lateral nucleus is a crucial gateway into the amygdala for the conditioned and unconditioned stimuli during the formation of fearful memories^1,6,45^. We found that our lateral cluster was strongly connected with sensory areas including occipital, parietal, tectum, and posterior temporal areas^17^. These connections have been implicated in fear recognition^16^. This lateral cluster also had stronger connectivity through the uncinate fasciculus with the PFC. On the other hand, the basal nucleus of the amygdala receives input from the lateral nucleus but also receives other regulatory signals from the PFC, cingulum, and hippocampus. These regulatory signals provide the contextual information to modulate the sensory information. In this sense, it has been suggested that the PFC and the hippocampal connections with the basal nucleus are involved in fear extinction^46,47^. Our findings revealed connections of the basal cluster with the PFC, hippocampus, and cingulum bundle. Finally, the centromedial nuclei has been implicated in translating this sensory and regulatory information from the lateral and basal amygdala to the hypothalamus, septal area, BNST, and midbrain dopaminergic neurons^7,42,43,48^. These connections are crucial in mediating autonomic and behavioral responses to a given stimuli^33^. Our findings revealed that the medial and basal clusters had strong connectivity with the hypothalamus, septal area, and BNST through the VAF and ST that are the main output of the amygdala. We also found unique connections of the medial cluster with the VTA through the medial forebrain bundle and this circuit have a role in reward-related behaviors^1,17,33,41^.

Others have studied the segmentation of the amygdala using structural, diffusion, and functional MRI. Bach et al. used connectivity to parcellate the amygdala un two clusters highly connected with the OFC and the temporal pole^12^. Abivardi et al., extending on these findings, studied the connectivity of these two segments with the thalamus and the cortex^49^. They found direct connections between the amygdala (more pronounced from the basolateral complex) and the paraventricular nucleus and pulvinar of the thalamus. These authors also found that the basolateral complex (deep cluster) had greater connectivity with sensory areas, similar to our findings for the lateral cluster. On the other hand, they found that the centrocortical complex (superficial cluster) had greater connectivity with limbic and olfactory cortical areas. This is not in agreement with our findings since we found that our most medial cluster (centromedial complex of the amygdala) had stronger connectivity with hypothalamus, basal forebrain, VTA, and other brainstem nuclei. These discrepancies may be related with the different configuration of our cluster distribution that in turn is related with the cortical target regions we used for the connectivity analyses.

Saygin et al. used connectivity-based parcellation based on prior description of the histological segmentation of the amygdala^11^. This group later extended their findings using high resolution structural MRI to segment the amygdala independent of connectivity information, given that connectivity can be affected in some patient populations^40^. Even though the justification of the later study is sound, the histological segmentation of the amygdala would not take into account individual differences in connectivity gradients that may go beyond its histological configuration. For example, Sakai et al. using axonal labelling, demonstrated significant amount of overlap between the projections of the pallidum and cerebellum in the ventral thalamus regardless of its histological configuration^50^. Moreover, Behrens et al. showed a parcellation of the thalamus based on cortical connectivity^51^ that is not exactly in agreement with the histological configuration of the thalamus^52^. These findings suggest that parcellation based on connectivity may provide additional insights on how the brain connect with these structures beyond its histological configurations that may be useful for therapeutic targeting^51,53^. For instance, the thalamic connectivity-based parcellation has been used to understand the effects of thalamic stimulation^54–57^. Also, the striatal parcellation has been used to analyze the effects of electrical stimulation within these structures in patients with depression and OCD^58,59^.

The current study has several limitations. These include the well-known tractography and registration limitations that have been described elsewhere^60–62^. Moreover, we cannot infer new findings with regards to the amygdala circuitry because tractography cannot discriminate between direct and indirect connectivity and cannot ascribe the directionality of connections. The amygdala region is prone to susceptibility artifacts, but the high-quality acquisition of the HCP and NKI imaging protocol could minimize these artifacts. Minimal distortion-related issues could still have affected our results. To compensate for that, we carefully inspected each DWI acquisition and excluded data that contained significant distortions. Moreover, we were not able to find significant differences in the connectivity of some of the brain regions with the clusters and this may be related with either a biological phenomenon or a methodological limitation. This could also be explained by the fact that there are many intra-amygdala connections that may not be resolved by tractography. Even though we used distance correction for the tractography process, distance-related bias is still a limitation and the intensity obtained with distance correction can be hard to interpret. Another limitation is the limited resolution of the DWI to further segregate smaller connections of the amygdala may have played a role in this limitation. This was the reason behind selecting no more than three clusters to consider the limited resolution of the DWI acquisition.

Clinical interventions have demonstrated the role of the amygdala in several behavioral and psychiatric disorders^5,8,45,63–65^. The behavioral effects of these interventions have shed light on the understanding of the amygdala network and how modulation of specific subregions within this nuclear complex can produce a broad range of positive and negative clinical effects^63,64,66–69^. The resolution of the conventional imaging studies, however, does not allow us to recognize the specific points associated with different behavioral outcomes. Therefore, we anticipate that the amygdala parcellation based on connectivity with areas related to behavior could be useful to understand the effects of brain interventions directed toward the amygdala circuit. The detailed connectivity of this region would also help to understand the circuitry of the amygdala and to find nodes within the network to potentially modulate for therapeutic purposes. More studies are needed with precise anatomical description of the circuits in a patient-specific manner to identify specific areas associated with benefit in patients with psychiatric conditions.

## Conclusions

We parcellated the amygdala based on its connectivity and mapped the amygdala circuit based on its functional role in behavior. Although, the configuration of the amygdala in this study corresponded well with histological findings, this parcellation provides an in vivo mapping of the human amygdala circuitry that correlates with the established behavioral model of the amygdala. The amygdala circuitry that we found in this study describes the basal and lateral segments as the main input area of sensory and regulatory information and the basal and medial segment as the main output area to the hypothalamus, brainstem, septal area and BNST. Given the important role of the amygdala in behavioral disorders, these results provide an important step towards patient-specific in vivo mapping of the amygdala circuitry to help guide neuromodulatory therapies.

## Data Availability

Derived and supporting data are available from the corresponding author upon reasonable request. The amygdala parcellation derived from this work is available at https://identifiers.org/neurovault.collection:9279.
