# Author Correction: Connectivity-based parcellation of the amygdala and identification of its main white matter connections

**DOI:** 10.1038/s41598-023-32200-8

**Published:** 2023-04-04

**Authors:** Josue M. Avecillas-Chasin, Simon Levinson, Taylor Kuhn, Mahmoud Omidbeigi, Jean-Philippe Langevin, Nader Pouratian, Ausaf Bari

**Affiliations:** 1 Department of Neurosurgery, University of Nebraska Medical Center, 988437 Nebraska Medical Center, Omaha, NE 68198-8437 USA; 2 David Geffen School of Medicine, University of California Los Angeles, Los Angeles, CA USA; 3 Department of Psychiatry and Biobehavioral Sciences, David Geffen School of Medicine at UCLA, University of California, Los Angeles, CA USA; 4 Department of Neurosurgery, David Geffen School of Medicine, University of California, Los Angeles, CA USA; 5 Neurosurgery Service, VA Greater Los Angeles Healthcare System, Los Angeles, CA USA; 6 Department of Neurological Surgery, UT Southwestern Medical Center, Dallas, TX USA

Correction to: *Scientific Reports* 10.1038/s41598-023-28100-6, published online 24 January 2023

The Acknowledgements section in the original version of this Article was omitted. The Acknowledgements section now reads:

“We would like to acknowledge the Casa Colina Hospital and Centers for Healthcare for their generous support of this work.”

The original Article has been corrected.

